# Purification and characterization of a novel ubiquitin-like antitumour protein with hemagglutinating and deoxyribonuclease activities from the edible mushroom *Ramaria botrytis*

**DOI:** 10.1186/s13568-017-0346-9

**Published:** 2017-02-22

**Authors:** Rong Zhou, Ya-Jie Han, Min-Hui Zhang, Ke-Ren Zhang, Tzi Bun Ng, Fang Liu

**Affiliations:** 1 The Key Laboratory of Molecular Microbiology and Technology, Ministry of Education, Nankai University, Tianjin, 300071 China; 2 School of Biomedical Sciences, Faculty of Medicine, The Chinese University of Hong Kong, Shatin, New Territories, Hong Kong, China

**Keywords:** *Ramaria botrytis*, Antitumour protein, Apoptosis, Hemagglutination, Deoxyribonuclease

## Abstract

A novel ubiquitin-like antitumour protein (RBUP) was isolated from fruiting bodies of the edible mushroom *Ramaria botrytis*. The protein was isolated with a purification protocol involving ion exchange chromatography on DEAE-Sepharose fast flow and gel filtration on Sephadex G-75. SDS-PAGE, Native-PAGE and ultracentrifugation analysis disclosed that RBUP was a monomeric protein with a molecular weight of 18.5 kDa. ESI–MS/MS demonstrated that it shared 69% amino acid sequence similarity with *Coprinellus congregates* ubiquitin (gi|136667). The protein exhibiting strong anticancer activity towards A549 cells. Analysis by employing AO/EB staining and Annexin V-FITC/PI detection indicated that the cytotoxic effect of RBUP was mediated through induction of apoptosis. Furthermore, RBUP displayed hemagglutinating and deoxyribonuclease activities. A temperature of 40 °C and pH of 7.0 were required for optimal DNase activity. Therefore, it was estimated that RBUP exerted its antitumour effect by inducing apoptosis, and its hemagglutinating and DNase activities were also thought to participate in this effect. These results demonstrated that RBUP was a multifunctional protein with potential medicinal applications.

## Introduction

Since the World Health Organization suggested that health care would be improved by integrating traditional and complementary medicines into the practices of health care service delivery and self-health care, increasing attention was attracted to edible mushrooms, which constitute an important part of TCM, because of the lower cytotoxicity and fewer side effects than chemical medicines.

*Ramaria botrytis*, a kind of edible mushrooms found all over the world especially in mountains of eastern Asia, Europe and North America, is well known for its peculiar flavor like chicken breast meat taste and a fruity scent (Bhanja et al. 2013). The fruiting bodies of *R. botrytis* have high medicinal values. In traditional Chinese medicine they are used to strengthen the stomach, subdue the adverse flow of Qi, dispel the wind, break blood and warm middle energizer. Hitherto, there has not been much research on *R. botrytis*. Components like minerals and amino acids (Chen and Xi 2001), small molecule compounds, e.g. phenolic acids and ceramide (Barros et al. 2009; Yaoita et al. 2007) and polysaccharides (Bhanja et al. 2014; Li et al. 2012; Wang et al. 2012), and bioactivities like antimicrobial activity (Alves et al. 2012), anti-oxidative activity (Barros et al. 2009; Kim and Lee 2003), anti-proliferation activity (Chung 1979), protective effect on liver injury (Kim et al. 2003; Kim and Lee 2003), and immunoenhancing activity (Bhanja et al. 2013) have been reported on the mushroom. However, no information about purified proteins from *R. botrytis* was available. Lee et al. tested the enzymatic activity in its water crude extract (Lee and Ha 2001).

In the present work, a pure novel ubiquitin-protein (RBUP) with antitumour, hemagglutination and DNase activities have been isolated and characterized from the dried fruiting bodies of *R. botrytis*. The protein with these activities were herein reported for the first time.

## Materials and methods

### Mushrooms, tumour cells and reagents

The edible mushroom *R. botrytis* was purchased in a local market in He’nan Province China. 293T (human embryonic kidney), HeLa (human sarcoma), A549 (human non-small-cell lung cancer), KB (human nasopharyngeal carcinoma), and MCF-7 (human breast cancer) cells were generous gifts from Professor Qiao (Nankai University, China). SRB, trypsin, AO, EB and Calf thymus DNA were from sigma. FBS was from Gibco. Annexin V-FITC/PI apoptosis detection kit and DMEM were from Gen-view Scientific Inc. DEAE-Sepharose and Sephadex G-75 were from GE Healthcare. All other chemicals used in this study were of analytical grade.

### Purification scheme

Dried fruiting bodies (3 kg) were ground and soaked in distilled water (10 mL/g). After extraction overnight at 4 °C, the homogenate was filtered and the filtrate was precipitated with 80% (NH_4_)_2_SO_4_ and then centrifuged. After the precipitate had been collected, re-dissolved, dialyzed and lyophilized, the crude protein (161,60 mg) was obtained. The crude protein was applied to a column of DEAE-Sepharose Fast Flow (2.0 × 40 cm). The adsorbed materials were eluted stepwise with 150 mM NaCl, 300 mM NaCl and 500 mM NaCl, respectively. The second adsorbed fraction D2 was pooled and then passed through a gel filtration Sephadex G-75 (1.0 × 55 cm) column. The homogeneity of the second fraction (SE2) was estimated by gel filtration on a Superdex 75 10/300 column by FPLC using an AKTA Purifier (GE Healthcare). The peak (SE2) obtained constituted a purified protein. It was named RBUP.

### Molecular weight determination

The purity and molecular weight of the protein RBUP were determined by both SDS-PAGE and ultracentrifugation. The molecular weight of the denatured protein was assayed in SDS-PAGE with a 15% resolving gel and a 5% stacking gel. At the end of electrophoresis, the gel was stained and the molecular weight of protein RBUP was estimated based on its electrophoretic mobility. A sedimentation velocity experiment was performed by using a Beckman/Coulter XL-I analytical ultracentrifuge at 60,000 rpm and 4 °C using absorbance detection and double-sector cells loaded with approximately 15 mM RBUP (Zhang et al. 2014). The data were analyzed by using the programs SEDFIT and SED-PHAT. Native-PAGE was carried out on ice using a 15% resolving gel and a 5% stacking gel as SDS-PAGE without SDS. The gel was stained with Coomassie brilliant blue.

### ESI–MS/MS for protein identification

The band of RBUP in SDS-PAGE was excised, destained and digested by a trypsin solution (70 μg/mL) at 37 °C overnight (Cui et al. 2014). Then the supernatant was collected and subjected to nanoelectrospray ionization followed by tandem mass spectrometry (MS/MS) in a Q Exactive (Thermo scientific) coupled online to the HPLC (Dionex) using a C18 column (5 μm, 150Å, Agela Technologies). Spectra were accumulated until a satisfactory S/N had been obtained. Parent mass peaks with the range from 350 to 2000 m/z were picked out for MS/MS analysis. MS/MS data were acquired and processed using Mass-Lynx V4.1 software (Waters, MA, USA) and were converted to PKL files by the Protein-Lynx 2.2.5 software (Waters, MA, USA). The PKL files were analyzed using the MASCOT search engine. The search parameters were defined as follows: database, SwissProt; taxonomy, Fungi; enzyme, trypsin; and allowance of one missed cleavage.

### Assay for antitumour activity

The antitumour effects of the purified RBUP on tumour cell lines were determined using the protein-staining SRB assay (Skehan et al. 1990). Different human cell lines, including 293T, HeLa, A549, KB, and MCF-7 were taken as targets for protein RBUP. The various cell lines were maintained in DMEM medium supplemented with streptomycin, penicillin and FBS at 37 °C in a humidified atmosphere of 5% CO_2_. 100 μL, cell suspension (1 × 10^5^ cells/ml), with cells in the exponential growth phase, were seeded into each well of a 96-well culture micro-plate. After incubation for 24 h, RBUP solution was added to yield final concentrations from 0 to 20 μM and incubation was continued for another 48 h. Sequentially, the cells were fixed in cold trichloroacetic acid (25 μL, 50%) and stained with 0.4% SRB solution. The protein bound dye was solubilized with 100 μL Tris–HCl (10 mM, pH 7.4) for determination of the optical density at 490 nm. The positive control was composed of cells treated with 100 μg/mL 5-fluorouracil (5-Fu). The vehicle control was composed of cells without any treatment.

$$ {\text{Cell viability }}\left( \% \right) = \left( {{\text{mean OD of treated cells}}/{\text{mean OD of vehicle treated cells}}} \right)\, \times\, 100\% $$

### AO/EB staining

In order to check the plasma-membrane permeability, the A549 cells were stained with AO/EB dye mixture (100 μg/mL acidine orange and 100 μg/mL ethidium bromide) (Tomar et al. 2014). In brief, 5 × 10^5^ cells were seeded in a 6-well plate. After incubating for 24 h, the cells were treated with serial concentrations of 0, 4, 12 and 20 μM RBUP for another 48 h. Then, 20 μL AO/EB dye mixture was added to the plate and the plate was observed under a fluorescent microscope (Olympus, Germany) 5 min later.

### Annexin V-FITC/PI staining

Cell apoptosis was determined by Annexin V-FITC assay (Hsin et al. 2015). A549 cells were incubated in a 6-well plate for 24 h, to which RBUP was added to yield a final concentration from 0 to 20 μM and the cells were incubated for a further 48 h. All cells were collected and subjected to Annexin V-FITC/PI apoptosis detection kit. Approximate 1 × 10^5^ cells were analyzed by utilizing flow cytometry (BD Biosciences). The apoptosis of A549 cells was expressed as a percentage of cells which were apoptotic.

### Assay for hemagglutinating activity

Hemagglutinating activity of RBUP was carried out as described by Li et al. In brief, RBUP was dissolved in phosphate-buffered saline (PBS, pH 7.0) and diluted in twofold series. Each 25 μL aliquot of the sample solution was mixed with 25 μL of the rabbit erythrocyte suspension (2%; v/v) in a microtitre U-plate at 37 °C. The agglutinating effect was recorded after about 1 h, when erythrocytes in the blank (PBS) had fully sedimented. The hemagglutination titer (one hemagglutinating unit) of a sample was expressed as the reciprocal of the highest dilution exhibiting visible agglutination of rabbit erythrocytes (Li et al. 2008).

### Assay for deoxyribonuclease activity

DNase activity was measured by a hyperchromicity assay (Baker et al. 1998). Calf thymus DNA (200 μg) from Sigma was incubated with the isolated protein in 500 μL 20 mM Tris–HCl buffer (pH 7.0). The reaction was terminated by introducing 500 μL of ice-cold perchloric acid (50%). After centrifugation, the absorbance of the supernatant measured at 260 nm was positively correlated to the deoxyribonuclease activity. The relative rates of DNA hydrolysis by RBUP at various pH values were determined with different buffers. Ammonium acetate buffer (20 mM) was used for pH 3–6 and Tris–HCl buffer (20 mM) for pH 7–10. The optimum temperature for the DNase activity was determined over the temperature range 20–90 °C. The effects of metal ions on DNase activity of RBUP were determined with the same hyperchromicity assay at pH 7.0 and 37 °C. In the test, the effects of divalent metal ions (MgCl_2_, MnCl_2_, ZnCl_2_, CaCl_2_, and CuCl_2_) and univalent metal ions (NaCl, KCl) were examined.

### Statistical analysis

Each experimental condition was analyzed at least three times in triplicate (n = 3). Data were expressed as the mean ± standard deviation (SD). Statistical analyses were carried out using one-way analysis of variance (ANOVA), followed by Tukey’s multiple-comparison test. Differences were considered statistically significant at *p < 0.05, **p < 0.01.

## Results

### Purification and molecular weight determination of RBUP

**Fig. 1:**
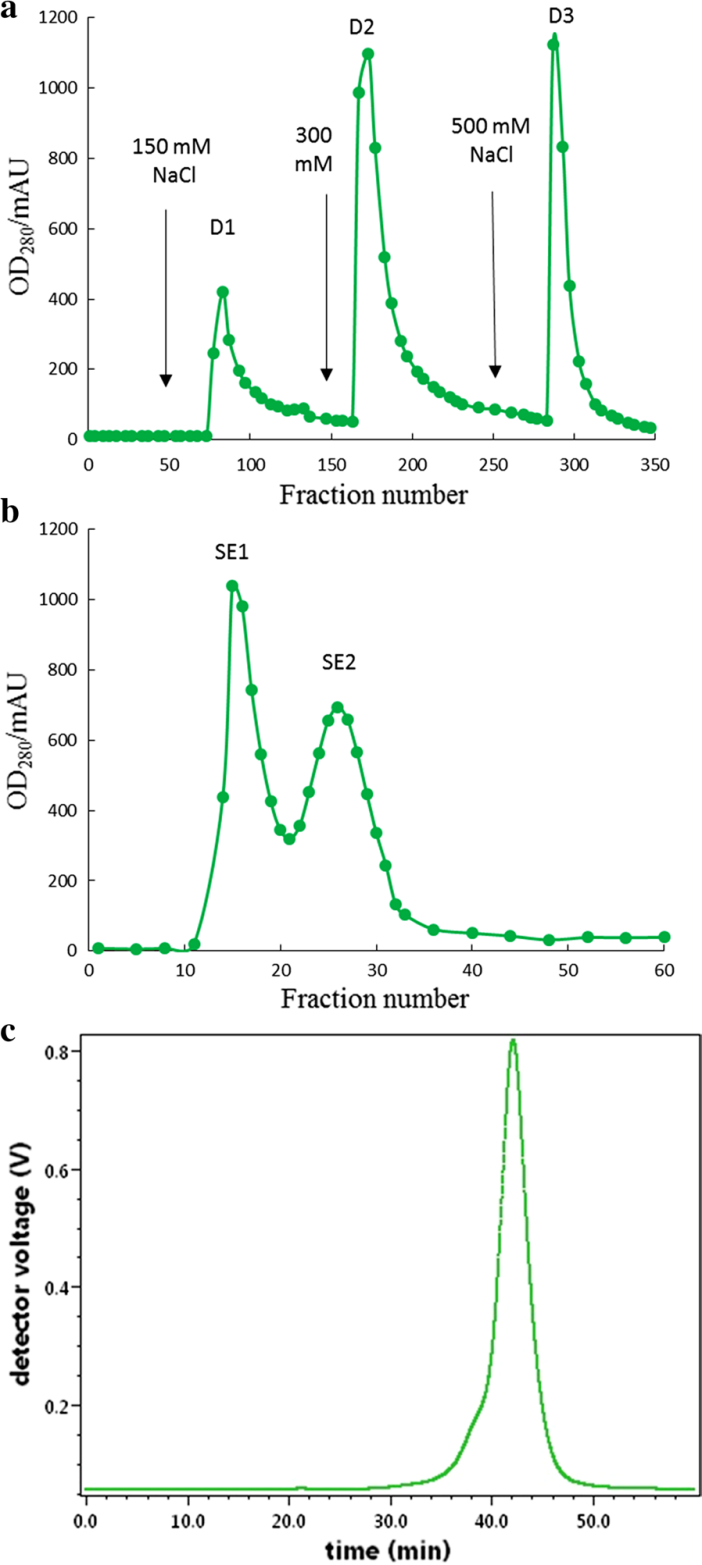
Purification of RBUP. **a** Crude protein eluted by anion exchange chromatography on DEAE-Sepharose. **b** Fraction D2 eluted by gel filtration chromatography on Sephadex G-75. **c** Fraction SE2 eluted from Superdex 75 by FPLC

**Fig. 2:**
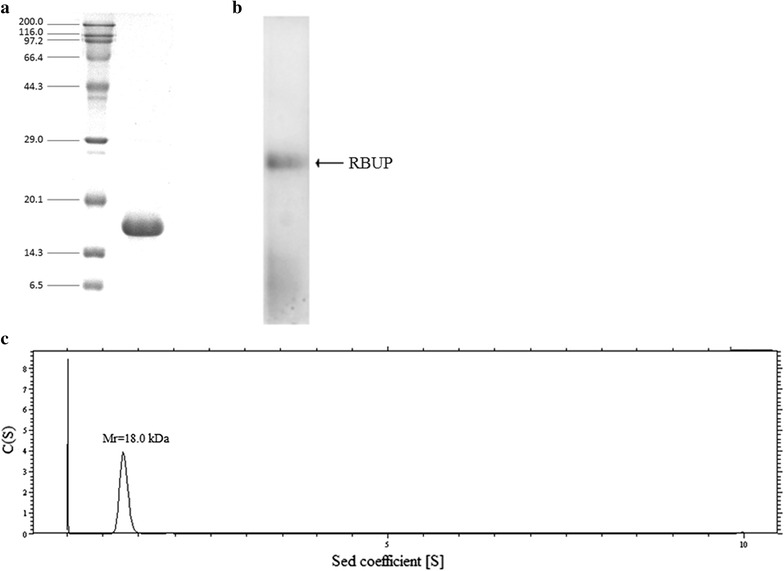
Molecular weight of RBUP. **a** SDS-PAGE. *Lane 1*, protein molecular weight markers; *lane 2*, purified RBUP. **b** Native-PAGE. **c** Ultracentrifugation

Anion exchange chromatography on DEAE-Sepharose Fast Flow resulted in three adsorbed fractions D1, D2 and D3 (Fig. 1a). Fraction D2 was pooled and concentrated. Subsequently, fraction D2 (3725 mg) was resolved into peak SE1 and peak SE2 through gel filtration on a Sephadex G-75 column (Fig. 1b). The homogeneity of the second peak (SE2, 1524 mg) was verified by FPLC on a gel filtration Superdex 75 10/300 column. A single peak was collected demonstrating that SE2 was purified (Fig. 1c). We named the purified SE2 fraction RBUP (*R. botrytis* ubiquitin-like protein). It was obtained with a yield of 9.43%. Furthermore, both the results of SDS-PAGE and native-PAGE after staining with Coomassie brilliant blue showed a single band, which revealed that RBUP was a monomeric protein (Fig. 2a, b). In an ultracentrifugation experiment, the sedimentation velocity can be monitored to calculate the molecular weight. Thus, ultracentrifugation was also used as an effective method to give the more precise molecular weight of RBUP. The results showed a single peak, and the molecular weight was calculated to be 18.5 kDa (Fig. 2c), which agreed with the results from SDS-PAGE. These results indicated that native RBUP was a monomeric protein with a molecular weight of 18.5 kDa.

### Protein identification

**Fig. 3:**
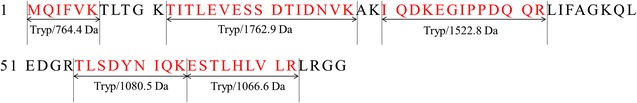
Peptide mass fragments of RBUP confirmed by a NCBI blast search of ESI–MS/MS. All *letters* stand for amino acid residues of *Coprinellus congregates* ubiquitin, the *red letters* stand for digested fragment of RBUP matched with *C. congregates* ubiquitin

ESI–MS/MS analysis and the currently known databases of proteins were combined to identify the sequence of RBUP. The results of mass spectrometry revealed that the protein RBUP possessed an amino acid sequence similar to *Coprinellus congregates* ubiquitin (gi|136667) confirmed by NCBI BLAST search (Fig. 3). The sequence coverage was 69%. The above results disclosed that RBUP was a novel ubiquitin-like protein.

### Antitumour activity

**Fig. 4:**
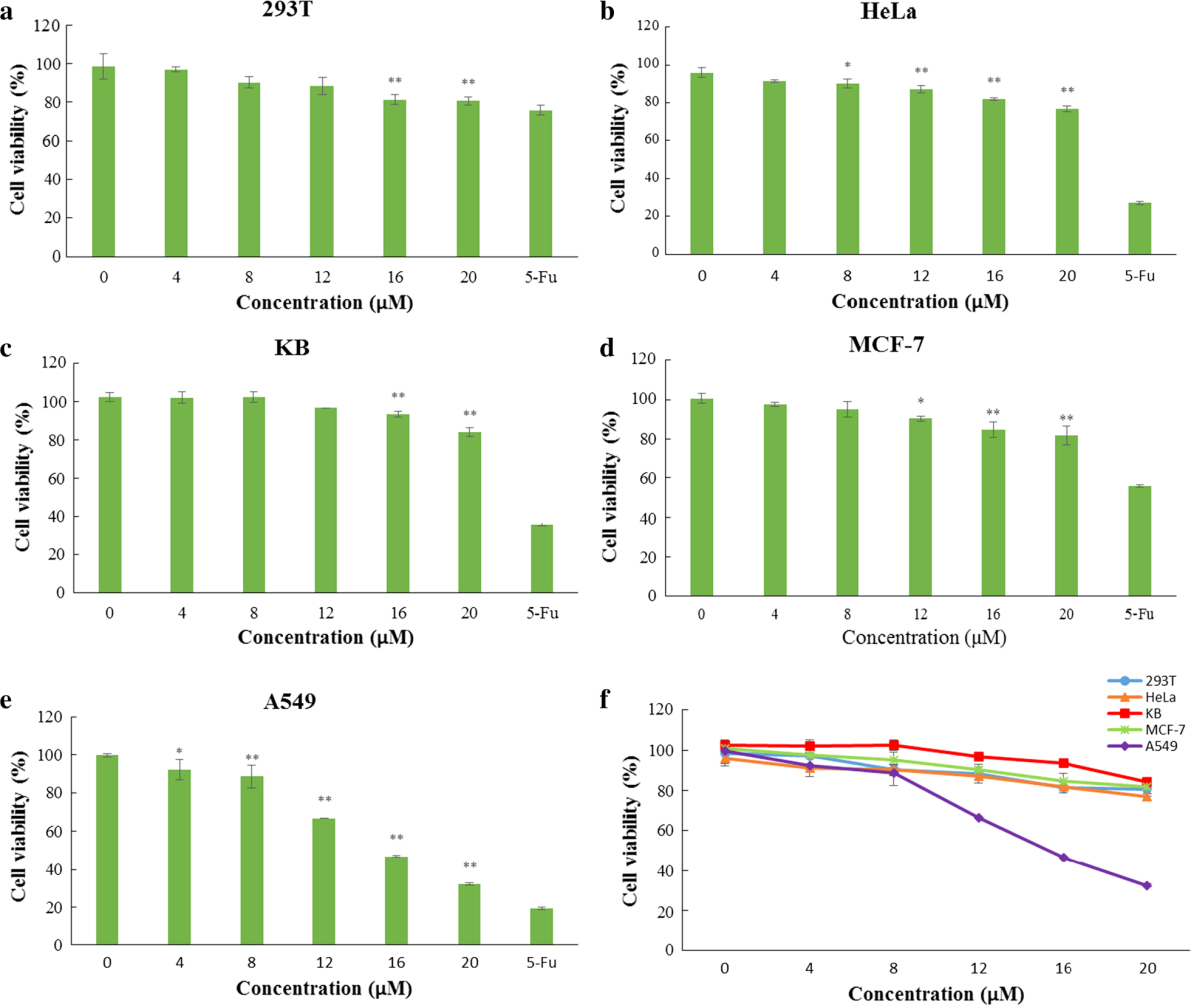
Antitumour activity on different cell lines. Histogram indicates the number of live cells in presence of different concentrations of RBUP. Symbol *asterisk* indicates p < 0.05, symbol *double asterisk* indicates p < 0.01

Figure 4a–f showed the growth inhibitory effects of RBUP at various concentrations against selected human cell lines including normal cells and tumour cells. Cell viability of normal human cells 293T treated with 20 μM RBUP was found to be 80.50%. It means that 20 μM RBUP did not cause significant damages to normal cells. So, the concentrations of 0, 4, 8, 12, 16 and 20 μM were chosen to examine the anti-tumour effects of RBUP towards Hela, KB, MCF-7 and A549 tumour cell lines. When the RBUP concentration was 20 μM, the cell viabilities of these tumour cell lines were 76.81, 84.12, 81.75 and 32.39%, respectively. Among all the cell lines tested, A549 cells were found to be most sensitive to RBUP where significant inhibition in cell viability was observed at 4 μM. RBUP exerted a significant dose-dependent antitumour effect towards A549 cells with an IC_50_ of 15.93 μM.

### RBUP induced apoptosis

**Fig. 5:**
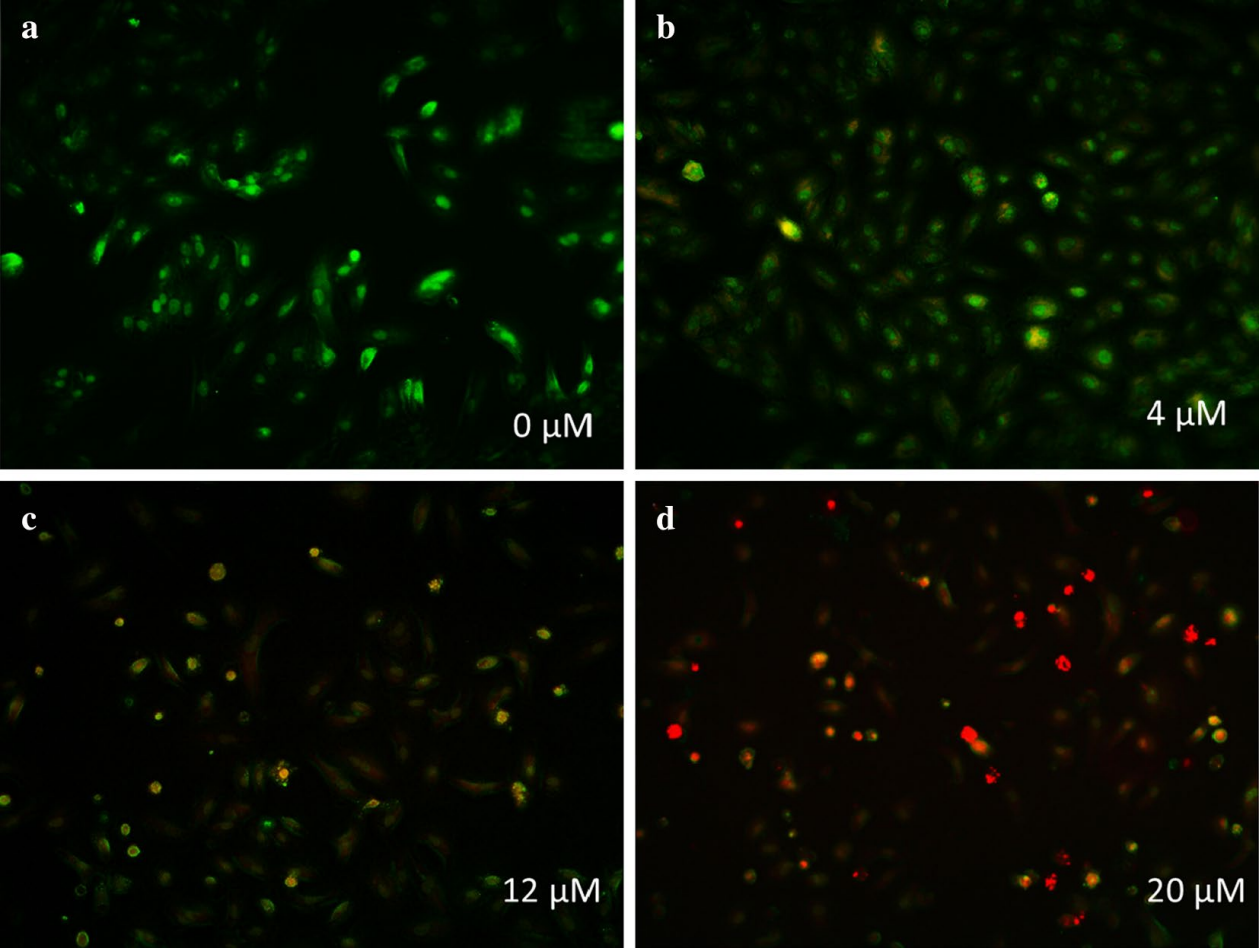
The effect of RBUP on A549 cells as observed by using AO/EB staining. The working concentrations were 0, 4, 12 and 20 μM

**Fig. 6:**
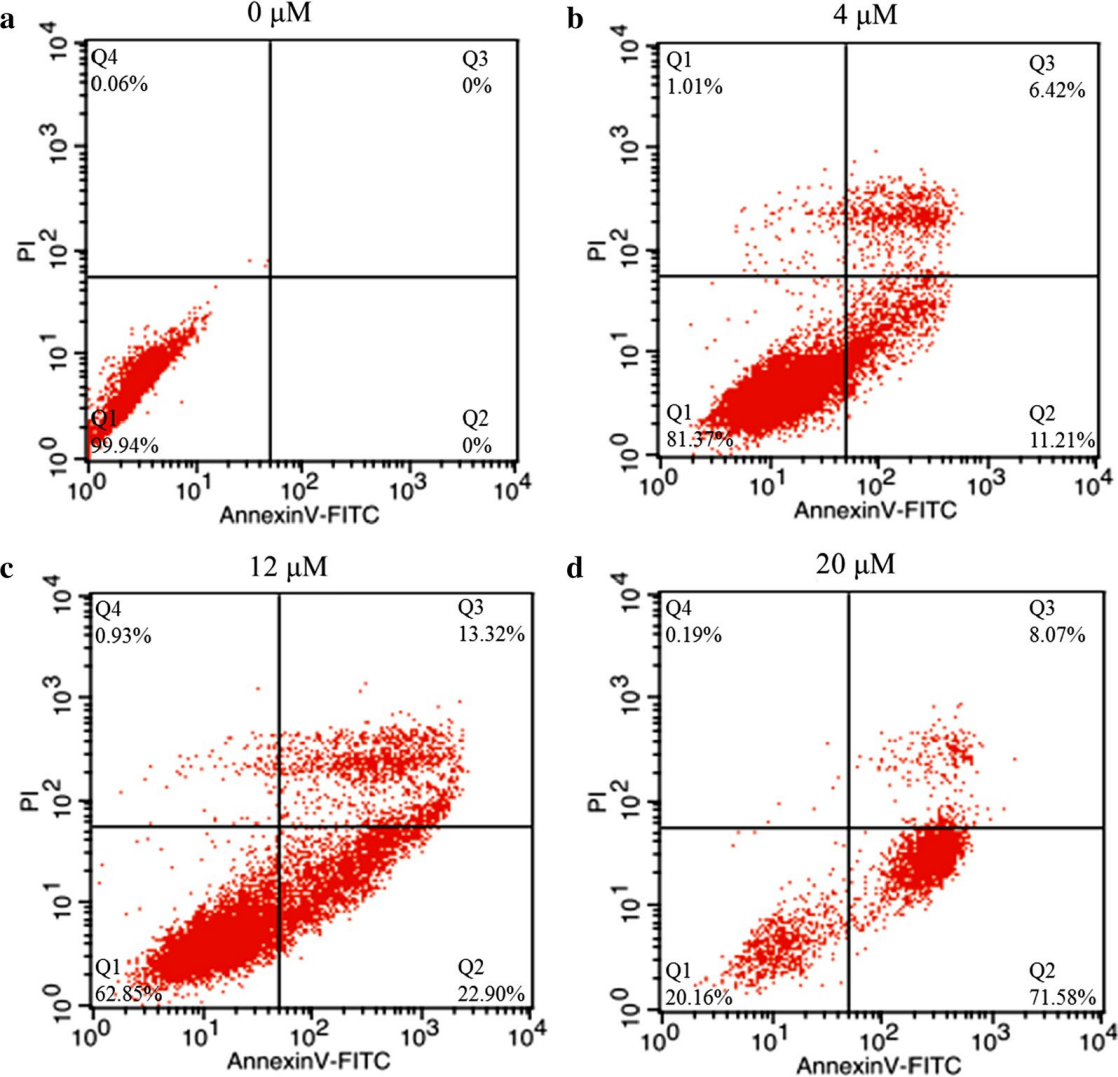
The effect of RBUP on A549 cells as observed by using FITC/PI staining. The working concentrations were 0, 4, 12 and 20 μM

In this research, AO-EB staining and Annexin V-FITC/PI apoptosis detection were employed to investigate the role of apoptosis in cytotoxicity of RBUP. As shown in Fig. 5a–d, the buffer treated cells (0 μM) had the maximum number of viable cells showing green color. On the contrary, in case of cells treated with RBUP, orange or even red cells appeared. When the concentration of RBUP was 4 μM, a few of the cells turned orangy–yellow. As the concentration of RBUP increased, the color of the cells turned orange and even red with the increasing percentage uptake of EB dye, indicating that apoptosis occurred in a dose-dependent manner. Flow cytometric analysis with FITC/PI staining is shown in Fig. 6a–d. The right quadrants of the figures represented the percentage of apoptotic A549 cells. The apoptotic rates were 0, 17.63, 36.22 and 79.65% respectively after incubation with RBUP at the concentrations of 0, 4, 12 and 20 μM. That indicated apoptotic cell death was prominent and took place in a dose-dependent manner. The results obtained from AO/EB and FITC/PI staining revealed that the protein RBUP induced apoptosis in A549 cells.

### Hemagglutinating activity

**Fig. 7:**
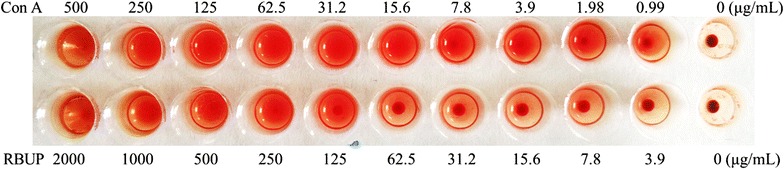
The hemagglutinating activity of RBUP towards rabbit red blood cells

Figure 7 showed that hemagglutinating activity of RBUP at 2.0 mg/mL was 32 U. When the concentration of RBUP was lowered to 250 μg/mL, erythrocytes were still agglutinated. Furthermore, red cells were totally sedimented while RBUP was at or below the dose of 62.5 μg/mL. However, RBUP exhibited a strong preference for rabbit red blood cells to mouse erythrocytes. It presented negative results in clotting experiments using mouse red blood cells (data not shown).

### Deoxyribonuclease activity

**Fig. 8:**
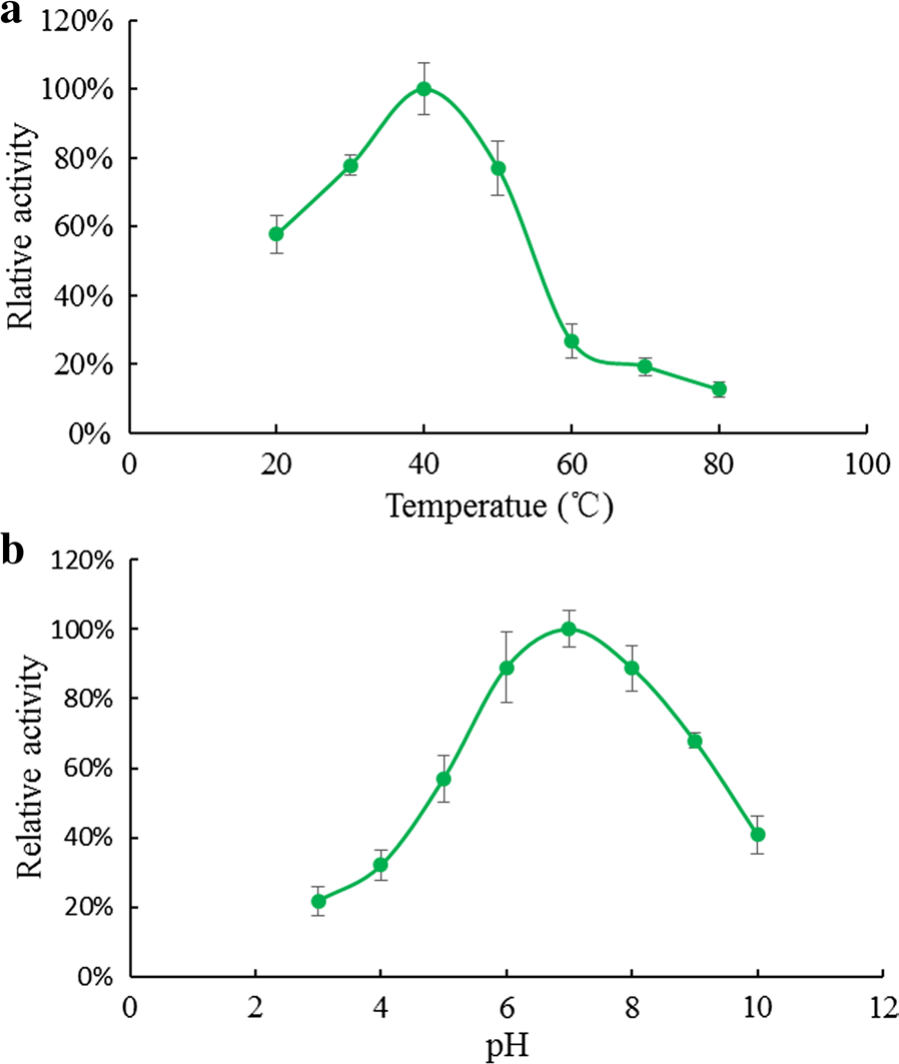
The effects of temperature and pH on DNase activity of RBUP

**Fig. 9:**
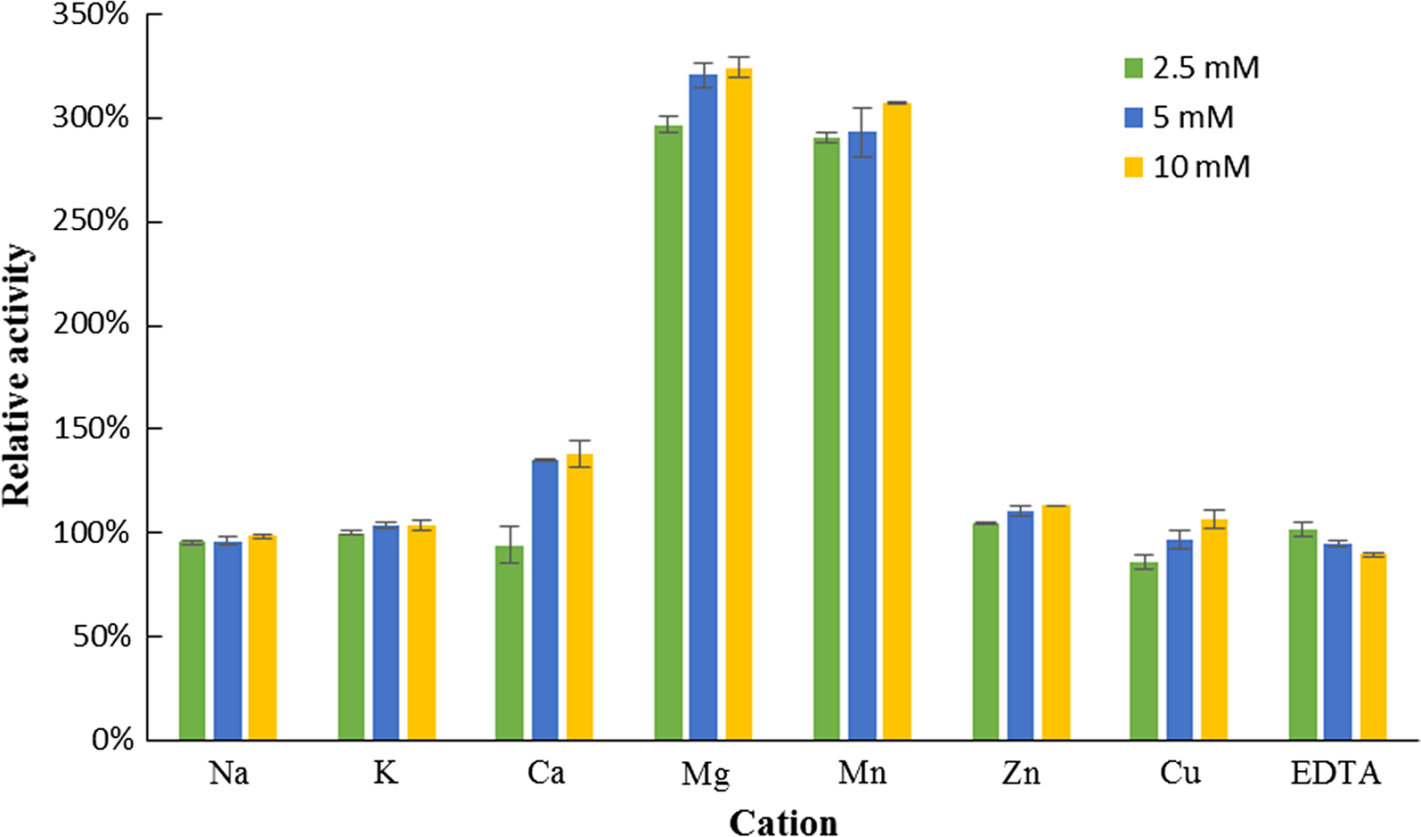
The effect of cations on DNase activity of RBUP

As shown in Fig. 8a, the deoxyribonuclease activity of RBUP was relatively stable between 30 and 50 °C. It expressed maximal activity at 40 °C. An increase in temperature beyond 40 °C ensued in a reduction of DNase activity. The activity decreased by almost 80% at 60 °C. The activity observed at 20 °C was considerably higher than that seen at 60 °C. High activity of the deoxyribonuclease was maintained at pH 6.0–8.0 with the maximum at pH 7.0 (Fig. 8b). Only 20% of the activity remained when the pH was 3.0. As shown in Fig. 9, the DNase activity of RBUP was dependent on divalent cations, but not dependent on univalent cations. The presence of Mg^2+^ and Mn^2+^ ions enhanced the DNase activity of this protein. The presence of Ca^2+^ marginally enhanced the activity. The monovalent cations, Na^+^ and K^+^ did not present significant effects on the activity. The DNase activity of RBUP was found to be not much sensitive to EDTA. 10 mM EDTA reduced only 10% of the DNase activity, but it could inhibit the hydrolysis when the concentration was increased to 100 mM. Our results showed that RBUP was a divalent metal ion-dependent, nonspecific DNase.

## Discussion

In the present study, a novel ubiquitin-like antitumour protein RBUP from *R. botrytis* with hemagglutinating and deoxyribonuclease activities was first identified, which will enhance its nutritional and medical applications and add to the meager knowledge about proteinaceous constituents of the mushroom.

The purification strategy for RBUP has some similarities with another protein from other *Ramaria* mushrooms. Both RBUP and *R. formosa* ribonuclease were adsorbed on DEAE column chromatography and unadsorbed on CM column chromatography (Zhang et al. 2015). Using the same purification scheme as that employed for RBUP, Zhao et al. purified an antitumour lectin AAL from the edible mushroom *Agrocybe aegerita*, which also possessed DNase activity (Zhao et al. 2003). PNAP, another antitumour protein with DNase activity from the edible mushroom *Pholiota nameko*, had different chromatographic characteristics. It was unadsorbed on anion exchange chromatography, but adsorbed on cation exchange chromatography (Zhang et al. 2014).

From the above results, we identified that RBUP was a monomeric 18.5-kDa ubiquitin-like protein. A range of low-molecular-weight proteins (below 20 kDa) from macrofungi have been reported to have potential antitumour activity. Among these proteins, nucleases take up a large proportion (Shuang et al. 2010; Wu et al. 2010; Zhang et al. 2010, 2014). Lectins (Marty-Detraves et al. 2004), FIPs (Chang et al. 2010; Liao et al. 2006; Lin et al. 2010b), RIPs (Lam and Ng 2001; Wong et al. 2008), ubiquitin-like proteins (Lam et al. 2001; Ngai et al. 2003), antifungal proteins (Chu et al. 2005), proteases (Sun et al. 2011) and hemolysins (Ngai and Ng 2006) belong to the remainder. As a new low-molecular-weight protein, RBUP also exhibited antitumour activity. Figure 4a–f showed that RBUP exhibited different antitumour effects against Hela, KB, MCF-7 and A549 tumour cells. Furthermore, based on the results of ESI–MS/MS, RBUP shared 69% amino acid sequence similarity with ubiquitin, which indicates possession of a highly conserved structure. The molecular weights of ubiquitin-like proteins with potential antiproliferative activity from *Calvatia caelata* and *Agrocybe cylindracea*are were 8 and 9.5 kDa, respectively, which were much lower than that of RBUP. Until now, RBUP is the isolated ubiquitin-like antitumour protein with the largest molecular weight.

Apoptosis is a kind of programmed cell death to maintain tissue homeostasis by removing extraneous or harmful cells. Apoptotic cells have characteristic cell changes, including blebbing, cell shrinkage, nuclear fragmentation, chromatin condensation, chromosomal DNA fragmentation, and global mRNA decay (Sanmartin et al. 2012). Recent studies have established that apoptosis plays an important role in cancer biology and is considered to be a primary mechanism against tumour cells. A convenient approach to evaluate cell apoptosis is AO/EB staining. The assay of AO–EB staining is based on cytoplasmic membrane integrity. In apoptotic cells the cytoplasmic membrane integrity is lost. It allows EB dye to enter the cells and stain the nucleus red. At the same time, AO dye permeates not only live cells but also apoptotic cells, and stains the nucleus green. So, the appearance of orangy cells indicates apoptotic cell death. As Fig. 5a–d showed, the number of cells, which were orange or even red, increased as the concentration of RBUP was elevated, indicating concentration-dependent apoptosis. On the other hand, in apoptotic cells, the membrane phospholipid phosphatidylserine (PS) is translocated from the inner to the outer leaflet of the plasma membrane, thereby exposing PS to the external cellular environment, which could be combined by Annexin V. An analysis which combines flow cytometry with Annexin V-FITC/PI mix dye could identify and quantify the percentages of live cells, apoptotic cells and dead cells. The points at the right upper and lower quadrants in Fig. 6a–d stand for apoptotic cells, those at left lower quadrant stand for normal cells, while those at left upper quadrant stand for PI positive cells (dead cells). These figures clearly revealed that the apoptosis-inducing potential of RBUP was concentration-dependent just like the effect of PNAP against MCF-7 cells (Zhang et al. 2014). However, the signal transduction of apoptosis induced by RBUP needs to be explored in greater detail.

Even though nearly 25 kinds of low-molecular-weight fungal proteins demonstrated antitumour activity, most of them have not been shown to inhibit growth of A549 cells. *C. caelata* ubiquitin-like protein (CULP) caused 50% cytotoxicity on MDA-MB-231 human breast carcinoma cells at 100 nM (Lam et al. 2001). *A. cylindracea* ubiquitin-like protein inhibited proliferation of M1 leukemia cells and Hep G2 hepatoma cells with the IC_50_ values of 10 and 100 μM, respectively (Ngai et al. 2003). The growth of MCF-7 cells was suppressed by RIP from *Hypsizigus marmoreus* (Wong et al. 2008), antifungal protein from *Cordyceps militaris* (Chu et al. 2005), and nucleases from *Lyophyllum shimeiji* (Zhang et al. 2010), *Pleurotus djamor* (Wu et al. 2010), *Russula delica* (Shuang et al. 2010), and *Pholiota nameko* (Zhang et al. 2014). Proliferation of Hela cells was inhibited by nuclease from *P. nameko* (Zhang et al. 2014) and lectin from *Xerocomus chrysenteron* (Marty-Detraves et al. 2004). The cytotoxicity of RBUP was higher to A549 cells than cytotoxicity to MCF, HeLa and KB cells, and it was below 20% towards 293T normal cells (Fig. 4a–f). The differential cytotoxic effects of RBUP can be of significance for medical use. Park et al. (2004) have reported that the different cytotoxicities of *Paecilomyces japonica* lectin (PJA) towards different cancer cell lines were related with its hemagglutinating activity, which was inhibited by sialic acid and sialoglycoproteins. The surface membrane expression of the sialoglycoconjugate structure in each of the cancer cell lines was the key to these differences. The hemagglutinating activity of RBUP may be associated with the differential cytotoxic effects. As Fig. 7 shows, RBUP exhibited lectin activity. PJA induced hemagglutinating activity in human ABO, mouse, rat, and rabbit erythrocytes. This was different from RBUP which agglutinated rabbit red cells instead of mouse red cells. What is more, PJA has little effect on tumour A549 cells. Hence, the different cytotoxic effects of RBUP could not be due to sialoglycoconjugate structure on the cell surface. A549 cells have been reported to have N-acetylglucosamine, d-galactosamine and N-acetylgalactosamine residues expressed on the surface (Xie et al. 2009). There is a need to further investigate whether N-acetylglucosamine, d-galactosamine and N-acetylgalactosamine are the sugars that exhibit specific binding to RBUP.

AAL, the mushroom lectin isolated by using the same purification strategy as RBUP, exerted its antitumour effect via apoptosis-inducing and DNase activities (Zhao et al. 2003). As Figs. 5a–d and 6a–d showed, RBUP could also induce apoptosis in A549 cells. In terms of molecular weight, molecular weight of PNAP is closest to that of RBUP. PNAP had been demonstrated to have DNase and antitumour activities as well (Zhang et al. 2014). Thus, DNA hydrolysis is a new direction to explore the bioactivities of RBUP. DNases are broadly divided into DNases I and II based on their optimal pH values and metal ion dependence (Cunningham and Laskowski 1953). Enzymes from DNase I family require a neutral pH and divalent cations, especially Mg^2+^ (Kishi et al. 2001). Class II DNases are a group of acidic enzymes that cleave DNA without requirement for divalent cations (Evans and Aguilera 2003). Our results showed that RBUP exhibited optimal activity at pH 7.0, and divalent cation was essential for its catalytic effect, indicating that RBUP belongs to DNase I family just like the antitumour protein (AAD) from *A. aegerita*, which was totally different from PNAP. However, there are still differences of metal ion dependence between AAD and RBUP. Both AAD and RBUP were strongly activated in the presence of Mg^2+^ and Mn^2+^ alone (Chen et al. 2012). The presence of Ca^2+^ marginally enhanced the activity of RBUP, whereas it did not influence that of AAD (Fig. 9). The divalent cation-dependence of DNase activity of RBUP is totally like that of pumpkin 2S albumin, which also exhibits its DNA hydrolytic activity non-specifically (Tomar et al. 2014). In addition, DNase I has been demonstrated to play a physiologically important part in apoptosis and has been proposed as an attractive candidate for cancer therapy (Shiokawa and Tanuma 2001). The DNA hydrolyzing effect may suggest a possible mechanism of the antitumour activity of RBUP.

It is generally known that the antitumour mechanism of lectins may be related with their immunomodulatory activity (Mody et al. 1995). Antitumour effect towards A549 cell lines was only exhibited by FIPs from *Flammulina velutipes* (Chang et al. 2010), *Ganoderma tsugae* (Liao et al. 2006) and *Ganoderma microsporum* (Lin et al. 2010a) among all low-molecular-weight proteins from mushrooms. As a protein with hemagglutinating activity and selectively inhibiting the growth of human tumour cells, it was essential to detect the immunomodulatory activity of RBUP.

In conclusion, a novel ubiquitin-like antitumour protein from the edible mushroom *R. botrytis* has been isolated and partially characterized in this report. The protein RBUP significantly inhibited the growth and induced apoptosis in A549 cells. The underlying mechanisms were related to its hemagglutinating and DNase activities. Confirming the antitumour activity of RBUP in vivo and determining the detailed mechanism should be required. Explaining the structure–function relationships and exploring its pharmacokinetics may lead to the development of a novel antitumour drug.

## Abbreviations

RBUP: *Ramaria botrytis* ubiquitin-like protein
SDS-PAGE: sodium dodecyl sulfate–polyacrylamide gel electrophoresis
ESI–MS/MS: electrospray ionization-tandem mass spectrometry
DNase: deoxyribonuclease
TCM: traditional Chinese medicine
SRB: sulforhodamine B
FBS: fetal bovine serum
DMEM: Dulbecco’s modified eagle medium
FITC: fluorescein isothiocyanate
PI: propidium iodide
FPLC: fast protein liquid chromatography
HPLC: high performance liquid chromatography
5-Fu: 5-fluorouracil
AO: acridine orange
EB: ethidium bromide
Con A: concanavalin A
FIPs: fungal immunomodulatory proteins
RIPs: ribosome inactivating proteins
PS: phospholipid phosphatidylserine
